# Leukaemia diagnosed as a consequence of haematuria assessment: a case report

**DOI:** 10.1186/1757-1626-0002-0000009289

**Published:** 2009-09-16

**Authors:** Sailaja Pisipati, Guy Lucas, Ian Pearce

**Affiliations:** 1 Department of Urology, Manchester Royal Infirmary, Oxford Road, Manchester, M13 9WL, UK; 2 Department of Haematology, Manchester Royal Infirmary, Oxford Road, Manchester, M13 9WL, UK

## Abstract

**Introduction:**

T-cell prolymphocytic leukaemia is a rare condition that constitutes around 2% of cases of small lymphocytic leukaemia in adults. It follows an aggressive clinical course with a poor prognosis.

**Case presentation:**

We report a 55-year-old male who was diagnosed with T-cell prolymphocytic leukaemia following investigations for microscopic haematuria. Splenomegaly was identified on a plain abdominal radiograph and intravenous urography, thus triggering further investigations and diagnosis.

**Conclusion:**

Our case-report serves to remind us of the need to bear in mind other possible pathologies outwith our own area of expertise when interpreting results of any kind. This is perhaps increasingly important in the current era of increasing sub-specialisation throughout medicine.

## Introduction

T-cell prolymphocytic leukemia (T-PLL) is a mature post-thymic T-cell malignancy with distinct clinical and laboratory features. T-PLL is a rare condition that constitutes around 2% of cases of small lymphocytic leukaemia in adults. It follows an aggressive clinical course with a poor prognosis. Median age is 65 years and it affects men slightly more often than women [1]. Main disease features at presentation are splenomegaly, lymphadenopathy, hepatomegaly, skin lesions, and marked lymphocytosis. There is no reported correlation between microscopic haematuria and T-PLL.

## Case presentation

A 55-year-old Caucasian male smoker with a history of industrial exposure to urothelial carcinogens was referred for assessment of microscopic haematuria and lower urinary tract symptoms. The patient admitted to feeling increasingly tired for over 6 months and had shingles in the distribution of the maxillary division of the right trigeminal nerve. Abdominal examination revealed fullness in the left hypochondrium and the left lumbar region.

Flexible cystoscopy revealed normal bladder urothelium. A control film of the intravenous urogram failed to demonstrate any areas of abnormal calcification but revealed a soft tissue shadowing in the left hypochondrium extending down to the left iliac crest. Subsequent contrast films of the IVU demonstrated bilateral prompt nephrograms with normal sized renal outlines and normal pelvicalyceal filling bilaterally. The left renal pelvis was, however, pushed medially and the pelvicalyceal system compressed by an extrinsic mass which was thought to represent an enlarged spleen. A renal ultrasound scan performed as part of investigations for microscopic haematuria, revealed normal anatomy and architecture of the kidneys, but no comment was made regarding any other intra-abdominal organs.

Full blood count demonstrated leucocytosis (WBC 351 × 10^9^/L) with a predominance of lymphocytes, macrocytic anaemia (Haemoglobin - 8.3 g/dl, MCV - 117 fl) and thrombocytopenia (Platelet Count 39 × 10^9^/L). Spherocytes, smear cells and red cells with polychromasia were identified on peripheral smear. A diagnosis of T-cell prolymphocytic leukaemia was established following confirmation on bone marrow aspirate. He was treated with a 12 week course of CAMPATH chemotherapy.

## Discussion

Although haematuria has been reported with bone marrow transplantation for leukaemia, there is no reported correlation between microscopic haematuria and leukaemia itself; neither is there a reported correlation between lower urinary tract symptoms and T-PLL. This association in our patient is merely a coincidental finding. 30% of patients with chronic lymphocytic leukaemia develop shingles (varicella zoster infection) at some stage during their illness [2]. However, the identification of the soft tissue shadow on the KUB and IVU which triggered further investigations to establish the underlying diagnosis, was of crucial importance. This highlights the significance of basic radiographic interpretation skills and serves to remind us of the need to bear in mind other possible pathologies outwith our own area of expertise when interpreting results of any kind. This is perhaps increasingly important in the current era of increasing sub-specialisation throughout medicine.

## Conclusion

There is no known association between haematuria or lower urinary tract symptoms and T-PLL. It is, however, crucial to analyse and interpret the available information in its entirety. Our case-report serves to remind us of the need to bear in mind other possible pathologies outwith our own area of expertise when interpreting results of any kind. This is perhaps increasingly important in the current era of increasing sub-specialisation throughout medicine.

**Figure 1:**
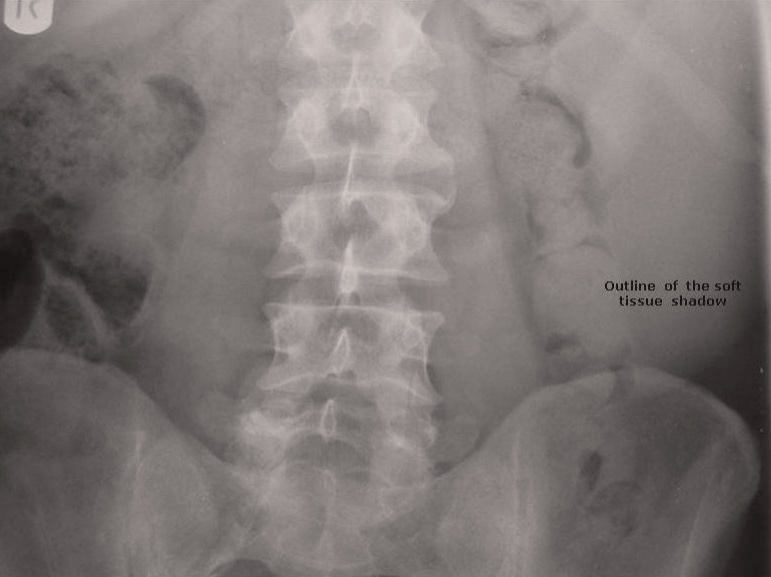
**Control film of the IVU demonstrating a soft tissue shadow in left hypochondrium and left lumbar region**.

**Figure 2:**
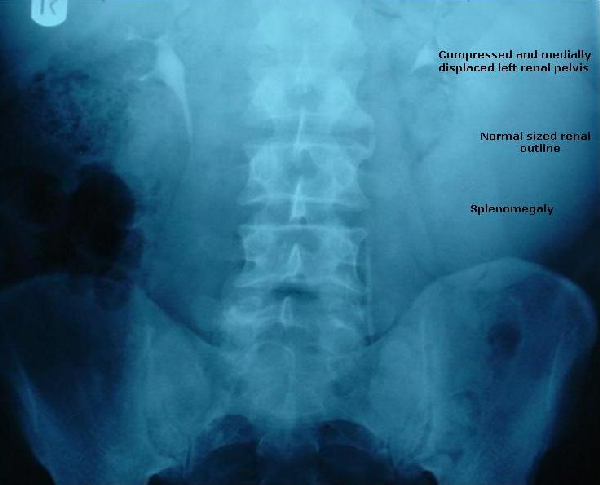
**Contrast film of the IVU**. Normal sized renal outline bilaterally. The left renal pelvis is compressed and medially displaced by an enlarged spleen.

## Abbreviations

IVU: intravenous urography; KUB: plain abdominal radiograph; MCV: mean corpuscular volume; T-PLL: T-cell prolymphocytic leukaemia; WBC: white cell count.

## Consent

Written informed consent was obtained from the patient for publication of this case report and accompanying images. A copy of the written consent is available for review by the Editor-in-Chief of this journal.

## Competing interest

The authors declare that they have no competing interests.

## Authors' contribution

IP investigated the patient for microscopic haematuria, identified the soft tissue shadow on plain radiograph and IVU and organised for haematological referral. GL organised for further investigations, established the diagnosis of T-PLL and commenced the patient on appropriate chemotherapy. SP was a major contributor in preparing the manuscript and obtained patient consent. All authors read and approved the final manuscript.
